# Designing strategies to implement a blunt chest injury care bundle using the behaviour change wheel: a multi-site mixed methods study

**DOI:** 10.1186/s12913-019-4177-z

**Published:** 2019-07-08

**Authors:** Sarah Kourouche, Tom Buckley, Connie Van, Belinda Munroe, Kate Curtis

**Affiliations:** 1 Faculty of Medicine and Health, The University of Sydney Susan Wakil School of Nursing and Midwifery, Mallet St, Camperdown, NSW Australia; 2 Emergency Services, Illawarra Shoalhaven Local Health District, Wollongong Hospital, Crown St, Wollongong, NSW Australia

**Keywords:** Health plan implementation, Nursing, Emergency, Organisations, Thoracic injuries, Thoracic wall, Rib fractures, Behaviour change wheel, Theoretical domains framework, Knowledge translation, Multidisciplinary implementation

## Abstract

**Background:**

Blunt chest injury can lead to significant morbidity and mortality if not treated appropriately. A *blunt chest injury care bundle* was to be implemented at two sites to guide care.

**Aim:**

To identify facilitators and barriers to the implementation of a *blunt chest injury care bundle* and design strategies tailored to promote future implementation.

**Methods:**

1) A mixed-method survey based on the theoretical domains framework (TDF) was used to identify barriers and facilitators to the implementation of a *blunt chest injury care bundle.* This survey was distributed to 441 staff from 12 departments across two hospitals. Quantitative data were analysed using SPSS and qualitative using inductive content analysis.

2) The quantitative and qualitative results from the survey were integrated and mapped to each of the TDF domains.

3) The facilitators and barriers were evaluated using the Behaviour Change Wheel to extract specific intervention functions, policies, behaviour change techniques and implementation strategies. Each phase was assessed against the Affordability, Practicability, Effectiveness and cost-effectiveness, Acceptability, Side-effects or safety and Equity (APEASE) criteria.

**Results:**

One hundred ninety eight staff completed the survey. All departments surveyed were represented. Nine facilitators and six barriers were identified from eight domains of the TDF. Facilitators (*TDF domains*) were: understanding evidence-informed patient care and understanding risk factors (*Knowledge*); patient assessment skills and blunt chest injury management skills (*Physical skills*); identification with professional role (*Professional role and identity*); belief of consequences of care bundle *(Belief about consequences*); provision of training and protocol design (*Environmental context and resources*); and social supports (*Social influences*). Barriers were: not understanding the term ‘care bundle’ (*Knowledge*); lacking regional analgesia skills (*Physical skills*); not remembering to follow protocol (*Memory, attention, and decision processes*); negative emotions relating to new protocols (*Emotions*); equipment and protocol access (*Environmental context and resources*). Implementation strategies were videos, education sessions, visual prompt for electronic medical records and change champions.

**Conclusions:**

Multiple facilitators and barriers were identified that may affect the implementation of a *blunt chest injury care bundle.* Implementation strategies developed through this process have been included in a plan for implementation in the emergency departments of two hospitals. Evaluation of the implementation is underway.

**Electronic supplementary material:**

The online version of this article (10.1186/s12913-019-4177-z) contains supplementary material, which is available to authorized users.

## Background

Mild to moderate blunt force chest wall injury can cause significant morbidity and mortality if not treated appropriately [1–3]. Injuries can be simple bruising of the chest wall, or fractures to the ribs or sternum caused by falls, violence or road trauma [1]; with rib fractures being the most common chest injury [4]. Multidisciplinary and comprehensive interventions for patients with blunt chest injury have been demonstrated to improve outcomes [5, 6]. However, the implementation of interventions to the multidisciplinary emergency context can be challenging, requiring a more strategic approach [7].

**Fig. 1:**
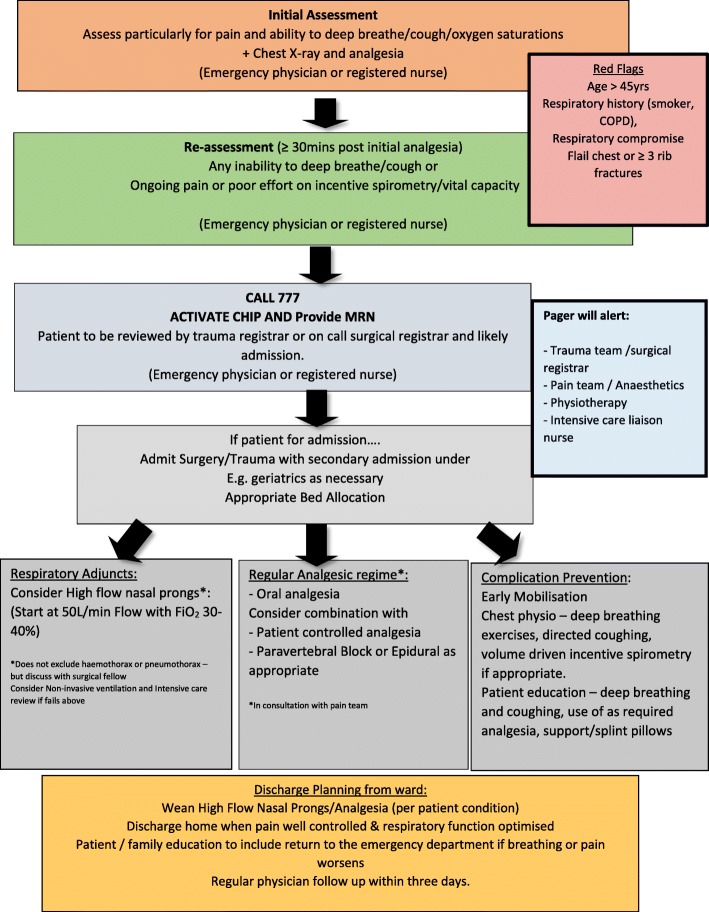
Algorithm for the *blunt chest injury care bundle* (Chest Injury Protocol - ChIP)

Recently, a “*blunt chest injury care bundle*” was developed to include a set of evidence-informed interventions for patients presenting to hospital with a blunt chest injury [8] (Fig. 1). A care bundle is a set of evidence-based interventions that when delivered together improve outcomes more than if they were administered separately [9]. This care bundle builds on a previous protocol piloted in St George Hospital [5, 7]. The care bundle now includes evidence-based interventions alongside role delineation for treating clinicians to facilitate consistent and evidence-based care [5, 7, 8]. Emergency clinicians (physicians or registered nurses) assess patients for eligibility and activate the care bundle. This then activates a pager system to alert the surgical or trauma team, physiotherapist, intensive care registrar and liaison nurse, and pain team to review the patient within 60 min [5]. The geriatric team can also be consulted if applicable. The effective implementation of this care bundle will require the staff involved to have the knowledge and skills to assess and manage a patient with blunt chest injury, identify a patient in need of admission and at potential for deterioration. Each of the different team members has a slightly different role that will affect the care of the patient and the implementation of the care bundle.

Change is already challenging in health care [10], the emergency setting can be more complex and challenging than many other acute areas with its multidisciplinary teams and quick paced environment [11]. However, there has been limited use of implementation research and theories to guide knowledge translation in the emergency setting [12, 13].

Clinician behaviour change is central to the success of knowledge translation [10]. The Behaviour Change Wheel is a framework that can help address the clinician behaviour change required for implementation to occur in the high acuity environment of the emergency department where there are multiple disciplines and departments involved [7, 14]. The framework provides step-by-step methods for identifying target behaviours for change to occur and then to develop strategies to target those behaviours [15], in line with the aims of this study. It is a thorough framework, designed from 19 frameworks of behaviour change from a systematic review [16]. The Behaviour Change Wheel provides the overarching framework used to identify the facilitators and barriers to the behaviours being targeted in implementation [16].

The Behaviour Change Wheel requires the user to define the problem in behavioural terms and select the behaviours to be targeted. The targeted behaviours required for the care bundle to be implemented were the *activation of the care bundle, responding to the care bundle activation* and *implementing the care bundle*. Within each of these three main areas more specific behaviours can be outlined; for example, the emergency medical practitioner or registered nurse would perform a thorough respiratory assessment of the patient, including their medical history and a chest x-ray. It was necessary to investigate whether these target behaviours were to be an area for change in the three target areas. A mixed-methods survey was used to identify the need for change (facilitators and barriers to change) and the information used to develop strategies of implementation.

This paper presents the prospective development of implementation strategies based on facilitators and barriers in a multidisciplinary emergency department context using the Behaviour Change Wheel. The specific aims of this study are:

1. To identify the facilitators and barriers to implementation of a blunt chest injury care bundle at two healthcare sites using a theoretical framework
2. To develop implementation strategies needed to inform an implementation plan based on the facilitators and barriers identified using a theoretical framework

## Methods

**Fig. 2:**
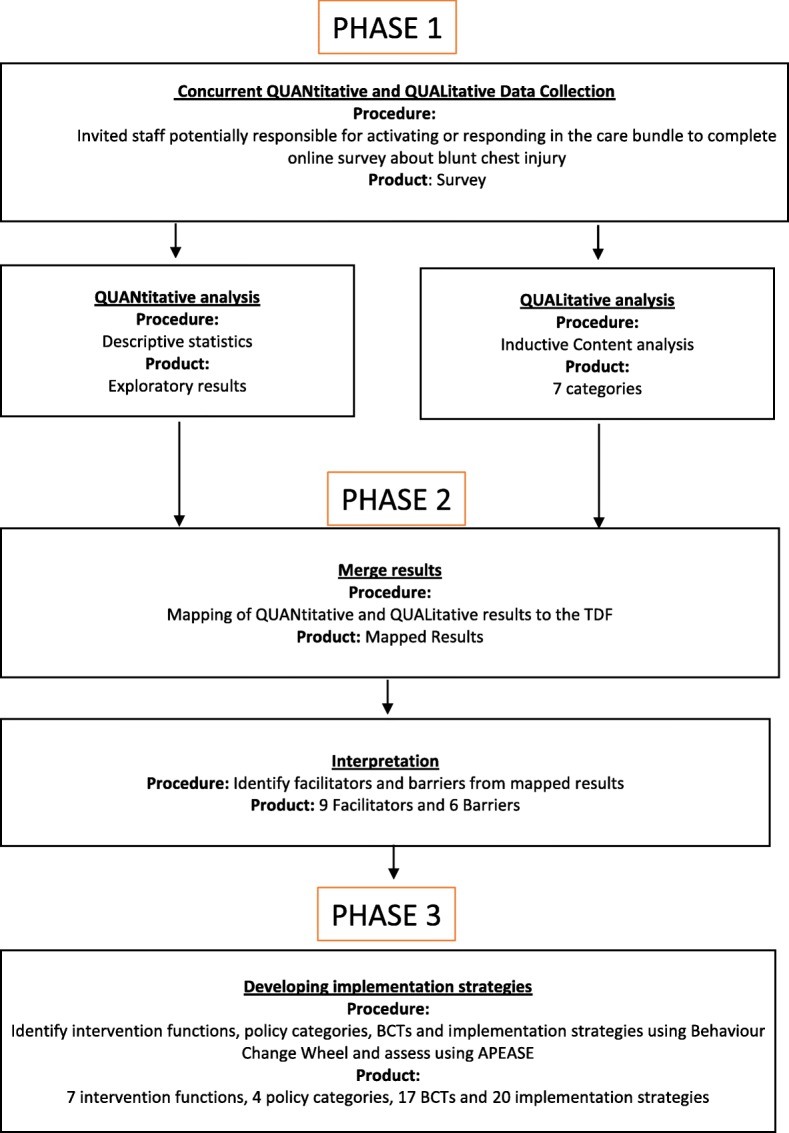
Study design showing the three phases

There are three phases to the study (Fig. 2): 1) concurrent quantitative and qualitative data collection via a mixed-method survey; 2) integration and interpretation of data to identify facilitators and barriers, and 3) the development of implementation strategies by mapping to the Behaviour Change Wheel.

### Phase 1: mixed-methods survey

#### Study design

A survey was used to identify facilitators and barriers to the introduction of a new care bundle at two hospital sites. The survey was a concurrent mixed-method survey with a quantitative focus and corresponding qualitative component [17, 18]. The use of quantitative data was used to identify facilitators and barriers. The corresponding qualitative component allowed for a further understanding of quantitative facets and also allowed the expression of areas not questioned in the quantitative section [17]. Following ethics approval, participants were recruited for six weeks between May and June 2017 from two Australian hospitals planning to implement the care bundle (Fig. 1).

#### Study sites

Two hospitals in the Illawarra Shoalhaven Local Health District (ISLHD) area south of Sydney, Australia were included in the study. The Wollongong Hospital is a 500-bed regional trauma centre, and the Shoalhaven District Memorial Hospital is a 200-bed local hospital.

#### Sampling and recruitment

Clinicians and staff potentially responsible for activating, responding or participating in the care bundle were invited to participate in the study. These are the staff that would potentially need to change their behaviour in response to the implementation of the care bundle. This included emergency registered nurses and medical practitioners, surgeons, anaesthetists, physiotherapists, clerical support staff, aged care and the pain team. There were an estimated 441 potential participants across 12 departments with a range of seniority, from junior medical officer to consultant.

Survey participants were emailed a link to the survey by the managers in the departments of surgery, anaesthesia, trauma service, physiotherapy, emergency department (ED) and the medical workforce. Surveys were completed anonymously online via REDcap (Research Electronic Data Capture) [19], a secure web-based application for data management and survey delivery. To improve participation numbers, participants were offered a movie ticket voucher as an incentive to complete the survey [20]. Reminder emails were sent every two weeks, and staff were allowed time during their shifts to complete the survey [21].

#### Instrument

The authors developed a 25-item questionnaire based on an integrative review on best-practice for patients with blunt chest injury [8] and theoretical frameworks for behaviour change. The Behaviour Change Wheel theorises that for behaviour change to occur persons involved need to have capability, motivation and opportunity to do so [22]. These broad fields are examinable in more detail using the Theoretical Domains Framework (TDF); which has 14 domains that have been linked to the capability, motivation and opportunity fields of the Behaviour Change Wheel.

The TDF is a validated framework of a synthesis of behavioural change theories for the assessment of implementation and behavioural change [23]. Using the TDF allows for a more comprehensive investigation of the range of facilitators and barriers to change [24].

Questions in the survey were based on each of the 14 TDF domains and the targeted behaviours identified relevant to the implementation of the *blunt chest injury care bundle* (Fig. 1)*.* The survey was adapted from previous similar questionnaires based on the TDF, one of which was validated [5, 25]. Questions about the knowledge and skills domains were informed by an integrative review of the interventions for blunt chest injury [8]. Questions surrounding the emotion domain was assessed using specific questions of interest adapted from Spielberger’s State-Trait Anxiety Inventory (STAI) Anxiety-State Scale [26]. This was to assess participant potential anxiety relating to the initiation of a new care bundle. The STAI Anxiety state scale was designed to assess anxiety in normal adults and has been tested extensively for reliability and validity [27]. For the complete survey see Additional file 1.

The questions were assessed for face validity by three doctoral trained nurses with content expertise. The questions were targeted explicitly to staff groups, for example, clerical staff were not asked questions relating to the clinical management of patients, and only medical practitioners were asked questions relating to the prescription of epidural analgesia. Responses to each item were with either a four, five or six-point Likert scale [28]. For example, “How important are the following interventions in the management of patients with blunt chest injuries?” with items such as “Early analgesia” and “Regular chest physiotherapy” on a 1–5 scale of not important, slightly important, moderately important, important, and very important. Open-ended questions were used after 14 of the quantitative questions enabling free text responses to collect qualitative data.

The survey was pilot tested with 12 medical practitioners, registered nurses and physiotherapists.

Items were both positively and negatively phrased to counter response set bias. The pilot group completed the survey on PC and mobile devices and provided feedback on the functionality of the survey, the time it took to complete, typographical errors found, questions irrelevant to their allocated role, and clarity of the instructions. Comments were collated, and changes made to the survey based on feedback.

#### Analysis

Quantitative and quantitative analysis for phase 1 occurred separately, and then the data was integrated in Phase 2 (Fig. 2).

##### Quantitative analysis

Quantitative survey data were analysed with descriptive statistics using SPSS IBM v24. Preliminary analyses were performed to ensure no violation of the assumptions of analysis including for normality and missing data. Responses were presented with mean (SD) or median (IQR) depending on distributions of data, or proportion data as appropriate.

##### Qualitative analysis

Qualitative data were analysed with NVivo 11 [29] following processes of inductive content analysis suggested by Graneheim and Lundman [30]. The responses were read through several times to get a sense of the whole and allocated into content areas [30]. The meaning units were developed by condensing the text. Meaning units were classified into categories and sub-categories. This process was completed by one author (SK); increasing the reliability of the interpretation by limiting the number of coders [2]. Any queries were resolved through discussion among three authors (SK, KC, TB).

### Phase 2: data integration and identification of facilitators and barriers

The quantitative and qualitative results from the survey were integrated and mapped to each of the TDF domains. Each item was reviewed to identify potential facilitators and barriers to implementation in each domain (authors SK, CV). Items were considered facilitators if: 1) they were positively worded with a mean or median greater or equal to four on a 5-point scale or equivalent, or 2) they were negatively worded with a mean or median less than or equal to one on a 5-point scale or equivalent. Items were considered barriers if they were positively worded with a mean or median less than three on a 5-point scale or equivalent or they were negatively worded with a mean or median greater than two on a 5-point scale or equivalent.

Higher mean scores represent stronger barriers and facilitators, and the researchers used this to assign a numerical target value [31–34]. From the qualitative data, codes that had three or more meaning units attached were flagged as potential facilitators or barriers. If it was unclear if an item was a facilitator or barrier for the implementation, then the consensus was sought from a senior member of the study team (KC) with more experience in use of the TDF. Any items that crossed multiple domains were resolved with a discussion between the authors and considered in the local context.

### Phase 3: development of implementation strategies

The facilitators and barriers from the survey were used to identify implementation strategies for behaviour change guided by the Behaviour Change Wheel. Authors SK, BM, KC conducted this phase. Stakeholders were involved in the assessment of intervention functions during a series of meetings where the initial concept was tabled at various departmental meetings to garner support for the intervention. Representatives from each stakeholder group were then consulted regularly through the implementation development process, for example, through a standing agenda item at several departmental meetings where the APEASE criteria guided the discussion. This was facilitated by two of the authors (KC, BM) who are emergency clinicians at the study sites and in regular direct contact with stakeholders.

Firstly, the authors used the facilitators and barriers identified in Phase 2 to identify intervention functions likely to be most effective. Intervention functions are “broad categories by means of which an intervention can change behaviour” [15]. The intervention functions were each assessed to see if they were Affordable, Practical, Effective and cost-effective, Acceptable, had Side-effects and were safe and Equitable (APEASE criteria) [15].

Secondly, the authors identified policy categories from the selected intervention functions and reviewed them using the APEASE criteria. Policy categories are groups of policy types that may aid intervention functions; these include legislation, guidelines or fiscal measures [28].

Thirdly, authors chose behaviour change techniques (BCTs) from the Behaviour Change Technique Taxonomy (BCTTv1) based on the intervention functions. A BCT is a component of an intervention that will alter behaviour [35]. The taxonomy includes 93 techniques for behaviour change linked to the Behaviour Change Wheel [35]. Each intervention function is associated with a list of BCTs that are relevant to that intervention function, which can be assessed for relevancy to the local context [4]. For instance, the implementation strategy for the BCT ‘Behaviour substitution’ would be that a clinician would substitute alcohol hand rub for washing with soap and water after attending to a patient assessment to improve hand cleaning compliance. Each BCT was also assessed using the APEASE criteria for inclusion.

Finally, the resulting BCTs were collated and were used to develop implementation strategies (modes of delivery) specific to the care bundle and context of the sites using information obtained from the survey.

## Results

### Demographics

The survey was completed by 198 participants (45% response rate) with a mean age of 37.6 years (SD 10.6) from 12 departments. All groups potentially impacted by the intervention implementation were represented. Sample demographics are presented in Table 1. Most participants were from Hospital A, the larger hospital, and worked in the emergency department.

**Table 1:** Demographics of participants completing the survey

	Participants (*n* = 198)	
Age in years mean (SD)	37.6	(10.6)
Gender (n, %)		
Female	112	57%
Male	85	43%
Hospital currently working (n, %)		
Hospital A	116	59%
Hospital B	72	36%
Both	10	5%
Clinical department and role (n, %)		
Anaesthetics (medical practitioners)	16	8%
Consultants /specialists	6	3%
Registrars/fellows	1	0.5%
Surgical (medical practitioners)	18	9%
Consultants / specialists	5	2.5%
Registrars/fellows	11	5.5%
Resident/interns	2	1%
Emergency Department	99	50%
Registered nurses	66	33%
Medical Practitioner	32	16%
Did not state	1	0.5%
Physiotherapy department	40	20%
Admissions department	10	5%
Other department specialist nurses	15	8%
Time in role (years) median (IQR)	5.0	(1.5–9.0)

### Phase 1: initial analysis of data

#### Quantitative data

Quantitative data generated from 25 survey questions are presented in Table 2. The results are further explained in phase 2 below linked to the facilitators and barriers.

**Table 2:** The facilitators and barriers identified linked to their TDF domains, with quantitative and qualitative results

TDF Domain	Factor affecting implementation	Facilitator (F) / Barrier (B)	Survey data			Qualitative data
Knowledge	Understanding of evidence-informed interventions for patient with blunt chest injury	F	How important are the following interventions in the management of patients with blunt chest injuries?	Median [IQR] (scale 1–5)^a^	Reponses (n)	Sub-category: Beliefs about blunt chest injury patient needs Example quote: “Takes an MDT [multidisciplinary team] to assess and manage these patients.”
			a. Early analgesia	5 [5–5]	171	
			b. Regular deep breathing and coughing	5 [4–5]		
			c. Maintaining oxygenation	5 [4–5]		
			d. Early mobilisation	5 [4–5]		
			e. Multimodal analgesia	5 [4–5]		
	Understanding of term care bundle	B	I am familiar with the term “care bundle.”	%	189	
			No	69.6		
			Yes	30.4		
	Understanding of blunt chest injury risk factors	F	Select the risk factors you feel are most likely to lead to deterioration for patients with blunt chest injuries	%	198	
			Elderly	80.3		
			3 or more rib fractures	87.9		
			COPD / Chronic lung disease	81.3		
Physical Skills	Confidence in patient assessment skills	F	In patients with blunt chest injury, I am confident in my ability to accurately….	Mean [SD] (scale 1–6)	168	
			a. Assess patient’s respiratory effort	5.38 [0.5]		
			b. Locate chest landmarks	5.26 [0.62]		
			c. Monitor for deterioration	5.11 [0.77]		
			d. Interpret findings from assessment of respiratory function	5.09 [0.76]		
			e. Describe findings from assessment of respiratory function	5.03 [0.73]		
			f. Assess pleuritic pain	4.86 [0.95]		
Physical skills (continued)	Confidence in skills needed for evidence-informed management of blunt chest injury	F	I am confident in my ability to accurately….	Mean [SD] (Scale 1–6)		
			a. Prescribe oral opioids (doctors/nurses)	5.35 [0.65]	130	
			b. Manage oral opioid analgesia (nurses)	5.33 [0.1]	64	
			c. Manage IV opioid analgesia	5.3 [0.7]	64	
			d. Prescribe appropriate analgesia	5.27 [0.77]	63	
			e. Monitor for deterioration	5.11 [0.77]	168	
			f. Set up high flow nasal cannula (HFNC)	5.33 [0.73]	54	
			g. Manage patient-controlled analgesia	4.91 [0.97]	64	
			h. Titrate flow rates for HFNC	4.88 [1.03]	130	
			i. Prescribe HFNC	4.84 [1.05]	55	
	Adequate skill in regional analgesia prescription and management	B	I am confident in my ability to accurately….	Mean [SD] (Scale 1–6)		Sub-category: Lack of experience. Example quote: “Skill levels for thoracic epidural/ paravertebral analgesia vary. A protocol will need to appreciate this or upskill a core group of clinicians to provide this service effectively”
			a. Prescribe epidural analgesia	3.57 [1.72]	63	
			b. Prescribe paravertebral block	3.56 [1.61]	55	
			c. Manage epidural blocks	3.52 [1.44]	64	
			d. Manage paravertebral blocks	3.03 [1.44]	64	
Memory, attention, and decision processes	Remembering to use protocol	B	In relation to experience with clinical protocols, I find it easy to remember when to activate new protocols	Mean [SD] (Scale 1–6) 4.32 [0.964]	176	Sub-category: Aids for implementation. Example quote: “…it is not easy to remember them [protocols] and they will get remembered wrong.”
Professional/ social role and identity	Identify with professional role associated with care of blunt chest injury patients	F	Relating to patients with blunt chest injury, it is my role to ...	Median [IQR] (Scale 1–5)		Sub-category: Staff roles. Example quote: “I have advocated for admissions for this patient group many times where the medical officer has felt the patient could be discharged.”
			a. Identify and escalate deterioration	5 [4–5]	153	
			b. Assess and recognise if need for further analgesia	5 [4–5]	153	
			c. Assess the patient	5 [4–5]	153	
Beliefs about consequences	Belief of consequences of care bundle	F	If a new protocol is implemented in your hospital, that activates an early multidisciplinary response (like a trauma call) and prompts evidence-based guidelines for patients with blunt chest injury. What statements reflect what impact you think it will have on you and/or your patient with blunt chest injury on the following?	Mean [SD] (Scale 1–6)		Subcategory: Optimism Example quote: “My previous experience with a [chest injury] protocol has been that it is easy to remember as it is used frequently enough that it becomes second nature and less protocol more a ‘reminder’ of what needs to be done”
			a. There will be overall improvement in patient care	5.18 [0.71]	148	
			b. The health care process will be improved overall	5.13 [0.70]		
			c. There will be improved time to physiotherapy review	5.05 [0.8]		
			d. There will be improvement in patient outcomes	Median 6 [5–6]	148	
			e. The patient will receive analgesia earlier	5 [5–6]		
			f. The patient will receive earlier pain team review	5 [5–6]		
			g. There will be improved time to medical review	5 [4–5]		
Emotion	Emotions relating to commencing new protocol	B	When using new protocols in my practice, I feel..........	Mean (scale 1–4)	160	Sub-category: negative feelings Example quote: “Doing a new task is challenging and inspiring but also anxiety producing as it is unfamiliar ground.”
			Positive related feelings – means ranged 1.99–3.05 [with SD 0.7–0.9]	2.71		
			Negative feelings – means ranged 1.15–1.47 [with SD 0.45–0.66]	3.04		
Environmental context and resources	Access to protocol	B	How likely are the following factors going to prevent you using protocols?	Mean [SD] (Scale 1–4)	166	Subcategory: System issues. Example quote: “…very hard to find protocols and guidelines online”
			a. Can’t find protocol when needed	3.11 [0.90]		
			b. No access to computer	2.63 [1.04]		
	Provision of training	F	How important are the following educational supports in using a new protocol?	Median [IQR] (Scale 1–5)	164	Subcategory: Recommended methods for education Example quote: “More face to face educational sessions”
			a. Help on the floor from senior staff	4 (4–5)		
			b. An educational session on the protocol	4 (4–5)		
			How likely is inadequate training in protocol going to prevent you using protocols?	Mean 3.05 [0.91]		
	The protocol design	F	How important are the following environmental factors in helping you remember to use clinical protocols?	Median {IQR] (Scale 1–5)	171	Subcategory: Protocol design Example quotes: “Succinct protocols are valued.” “The protocol has to be appropriate and rigorously tested”
			Simple criteria for activation of protocol	4 [4–5]		
			How likely is it that an unclear protocol is going to prevent you using protocols?	Median [IQR] 3 [2–4]		
	Access to equipment	B	How important are the following environmental factors in helping you remember to use clinical protocols?	Median [IQR] (Scale 1–5)		Subcategory: Equipment issues Example quote: “Access to PCA an issue”
			Having equipment easily accessible	4 [4–5]		
Social influences	Social Supports	F	I am more likely to follow a new protocol if I have support from.........	Median [IQR] (Scale 1–6)	164	Subcategory: Recommended issues for education Example quote: “All staff potentially involved in implementing a new protocol need to be included in all education for it to be successful, not just some disciplines”
			My superiors	5 (5–6)		
			Medical staff	5 (5–6)		
			Nursing staff	5 (5–6)		
			My colleagues	5 (5–6)		
			The patient	5 (4–5)	155	
			The patient’s family	5 (4–5)		

^a^Figures represent Likert scale range

#### Qualitative data

Four content areas were identified: (1) protocol use in the hospital environment, (2) care of blunt chest injury patient, (3) feelings towards implementation of protocols, and (4) practical advice for implementation. There were seven categories identified: implementation strategies; clinical needs of blunt chest injury patients; current issues in implementation; staff barriers to implementation; staff facilitators to implementation protocols; and roles (Table 3). These results are integrated with the quantitative data (Table 2).

**Table 3:** Content analysis of qualitative data from the survey presented in Categories and subcategories

Categories	Subcategories
Implementation Strategies	Aids for implementation Methods of communication Methods for education
Clinical needs of patients with blunt chest injury	Beliefs about patient needs Chest physiotherapy
Current issues in implementation	System Issues Equipment issues
Staff barriers to implementation	Lack of experience Negative emotions
Staff facilitators to implementation	Optimism Patient involvement
Protocol	Protocol design Protocol Benefits
Roles	Staff Roles

### Phase 2: interpretation of data to identify facilitators and barriers

There were nine facilitators and six barriers identified as impacting the implementation of the blunt chest injury care bundle. The facilitators and barriers were across eight domains of the TDF, including: ‘knowledge’, ‘physical skills’, ‘memory, attention and decision processes’, ‘professional/social role and identity’, ‘beliefs about consequences’, ‘emotion’, ‘environmental context and resources’ and ‘social influences’ (Table 2). No facilitators and barriers were identified in the other domains of the TDF. Facilitators and barriers are summarised in Table 2 and further explained within the text below.

#### Knowledge

There were two facilitators and one barrier within the *knowledge* domain (Table 2). Fifty-six per cent of clinician participants (i.e. not administration staff) responded they would know which treatment to initiate most of the time. The highest selected interventions for patients with blunt chest injury matched those supported by evidence to improve outcomes; indicating that participants had knowledge of evidenced interventions (facilitator). In the qualitative responses, respondents reported chest physiotherapy as an important treatment for patients with blunt chest injury though not with incentive spirometry (sub-category: Chest physiotherapy).

The majority of participants correctly identified the most significant risk factors for patient deterioration in blunt chest injury (facilitator – Table 2), including three or more rib fractures, chronic lung disease, and older persons over 65 years, indicating an understanding of blunt chest injury.

Participants demonstrated a poor understanding of the term care bundle (barrier), with only a small portion of respondents saying they understood the term (Table 2). However, 84% of those who said they did understand the term selected the correct definition, “A group of key evidence-based interventions for a specific condition”.

#### Physical skills

Participants reported confidence in blunt chest injury patient assessment and management (facilitators); however, they identified a lack of confidence in clinical skills relating to regional analgesia (barrier) (Table 2). Medical practitioners and registered nurses reported they lacked confidence in prescribing and managing epidural and paravertebral analgesia (Table 2).

#### Memory, attention and decision processes

In the *memory, attention and decision processes* domain, not remembering to use protocols was identified as a potential barrier to implementing the blunt chest injury care bundle (Table 2). When respondents were asked whether they thought there were too many protocols to remember, 63% reported this was true occasionally (42%) or most of the time (21%). The qualitative comments also reflected difficulty in remembering protocols, as one participant commented *“…I can’t remember all the Protocols”* (sic).

#### Professional/social role and identity

One facilitator was identified related to the *professional/social role and identity* domain. Clinical participants reported they had a professional responsibility towards patients with blunt chest injury, especially to assess and recognise if a patient with blunt chest injury needs further analgesia or is deteriorating (Table 2). Fifty-seven per cent of registered nurses did not agree that it was their role to decide if a patient needs admission; however, qualitative data indicated that they thought their role should be to advocate for the patient to be admitted if necessary (Sub-category: staff roles).

#### Belief about consequences

In relation to the *belief about consequences* domain, participants reported that they believed the *blunt chest injury care bundle* would result in the improvement of the health care process overall (Table 2). When asked about the impact that a protocol for chest injury may have they reported that it would be beneficial to patient outcomes, and patients would receive earlier analgesia, physiotherapy, medical and pain team reviews (Table 2).

#### Emotions

Participants reported mixed responses regarding the initiation of new protocols (Table 2). Most participants reported positive feelings of calm, security, comfort and ease with new protocols on the State – Anxiety items; however, the positive feelings did not score high enough to be considered a facilitator. Scores were low for the negative feelings of tension, anxiety and nervousness (Table 2).

One barrier was identified in the *emotions* domain. Qualitative responses indicated that some respondents had negative feelings towards new protocols. The negative feelings were reported to be due to fear of not following the protocol properly, or a lack of understanding of the protocol itself. Qualitative results suggested that some respondents thought new protocols could elicit negative feelings. For example, “*First time implementing a new protocol is always a bit nerve wrecking as you are explaining and trialling it for the first time*” (Sub-category: negative feelings). However, respondents reported that an improved understanding of the protocol reduces negative feelings. Respondents reported that they felt more confident in implementing a protocol when it was supported by evidence. An example quote from a participant was, “*I feel comfortable implementing protocols if they are well-researched, evidence-based and signed off by relevant experts pertaining to that particular protocol…*” (Sub-category: Optimism).

#### Environmental context and resources

There were two barriers and two facilitators identified within the *environmental context and resources* domain. Having equipment that is easily accessible and simple criteria for activation were reported as the most important environmental factors when it came to helping respondents remember to use protocols (Table 2). Email reminders to staff and department flyers were reported to be the least important (mean [SD] - 2.67 [1.2] and 3.11[1.1] respectively). Having an icon in the electronic medical record (medium 4 [IQR 3–5) or a checklist in the notes (medium 4 [IQR 3–4) were both identified as potential methods of improving respondents remembering to use protocols.

Participants identified that inadequate training would prevent protocol use and that they preferred face to face education rather than online learning (mean [SD] - 3.23 [1.6]). Receiving help on the floor from senior staff and receiving in-services on the protocol were the most popular choice for educational supports (85 and 76% indicated it was either important or very important respectively).

Qualitative data indicated it was important to participants that protocols they are expected to use be evidenced-based, easy to follow and easily accessible. Easy access to the protocol was considered particularly important (subcategory: aids for implementation). For example, one participant stated, *“Protocols need to be easy to find online or in a folder - it is not easy to remember them and they will get remembered wrong”.* Prompts and cues, such as checklists and flags, were also identified in the qualitative data as important environmental factors that helped participants remember to use protocols.

#### Social influences

One facilitator was identified in the *social influences* domain (Table 2). Support from colleagues (47%) and supervisors (45.1%) was reported as very important.

### Phase 3: implementation strategies selection

The authors identified nine intervention functions from the eight TDF domains. These were reduced to seven intervention functions after APEASE assessment, including *education, persuasion, incentivisation, training, environmental restructuring, modelling,* and *enablement* (Table 4). The intervention functions *coercion* and *restriction* were excluded after assessment with APEASE as *coercion* was deemed not ‘acceptable’ and *restriction* considered not ‘practical’, ‘effective’ nor ‘safe’.

**Table 4:** The eight TDF domains identified to contain facilitators and barriers (vertical) mapped to intervention functions (horizontal)

	Education	Persuasion	Incentivisation	Coercion	Training	Restriction	Environmental restructuring	Modelling	Enablement
Knowledge	✓								
Physical Skills					✓				
Memory					✓		✓		✓
Role	✓	✓						✓	
Beliefs about consequences	✓	✓						✓	
Emotion		✓	✓	✓				✓	✓
Environmental Context					✓	✓	✓		✓
Social Supports						✓			

The policy categories identified were *communication, guidelines, regulation, and environmental restructuring*. The policy categories *fiscal measures* and *legislation* were not included as they were both not ‘practical’ in this case as these are more suited to government or higher management-based initiatives.

Seventeen BCTs were identified (Table 5). One example of a BCT that was not included was ‘Self-monitoring of behaviour’ which required the staff member to monitor and record their behaviour. This was deemed not ‘practical’ on the APEASE as it was not feasible for staff to do as part of their workday. The resulting implementation strategies and modes of delivery are represented in Table 5.

**Table 5:** Behaviour change taxonomies (BCTs) with their relating intervention functions and planned implementation strategy specific to the care bundle

BCTs	Intervention Functions	Proposed implementation strategy	Barrier(s)/Facilitator(s) addressed (TDF domain)
Information about health consequences	Education, persuasion	Staff will be informed about the improvement in pneumonia rates reduction with the protocol from previous study	Belief of consequences of care bundle (Belief about consequences) Understanding of evidence-informed interventions for patient with blunt chest injury (Knowledge)
Feedback on behaviour	Education, persuasion, incentivisation	Staff compliance will be monitored through audits and by clinical champions Staff will be informed of the results informally via clinical champions and formally through email and newsletter correspondence	Belief of consequences of care bundle (Belief about consequences) Understanding of evidence-informed interventions for patient with blunt chest injury (Knowledge)
Feedback on outcome(s) of behaviour	Education, incentivisation, training	Feedback will be given to staff on patients treated with the care bundle	Understanding of evidence-informed interventions for patient with blunt chest injury (Knowledge) Belief of consequences of care bundle (Belief about consequences) Emotions relating to commencing new protocol (Emotion) Remembering to use protocol (Memory, attention, and decision processes) Confidence in patient assessment skills (Physical skills)
Information about others’ approval	Education, persuasion	Local staff will appear in the care bundle video showing support	Identify with professional role associated with care of blunt chest injury patients (Professional/ social role and identity)
Credible source	Persuasion	Senior local staff will appear in a video informing staff about the care bundle	Identify with professional role associated with care of blunt chest injury patients (Professional/ social role and identity)
Prompts/cues	Education, environmental restructuring	A visual prompt (screen icon) will be developed for the electronic medical record to flag to staff that patient is eligible for care bundle Flyers will be put up around the workplace to remind staff of the care bundle	Remembering to use protocol (Memory, attention, and decision processes)
Verbal persuasion about capability	Persuasion, enablement	Staff will be encouraged during educational sessions and by change champions that they are capable of following the care bundle	Emotions relating to commencing new protocol (Emotion)
Identification of self as role model	Persuasion, enablement	Staff will be asked to volunteer for the roles of change champions and to be in the video	Emotions relating to commencing new protocol (Emotion) Identify with professional role associated with care of blunt chest injury patients (Professional/ social role and identity)
Commitment	Incentivisation, enablement	Staff will appear in a video committing to the care bundle	Remembering to use protocol (Memory, attention, and decision processes)
Demonstration of behaviour	Training, modelling	Staff will receive demonstrations of behaviour in a video, in education sessions and at the bedside with the change champions	Confidence in patient assessment skills, Confidence in skills needed for evidence-informed management of blunt chest injury, Adequate skill in regional analgesia prescription and management (Physical skills) Remembering to use protocol (Memory, attention, and decision processes) Identify with professional role associated with care of blunt chest injury patients (Professional/ social role and identity) Emotions relating to commencing new protocol (Emotion)
Instruction on how to perform behaviour	Training	Staff will receive instructions of behaviour in a video, in education sessions and at the bedside with the change champions	Confidence in patient assessment skills, Confidence in skills needed for evidence-informed management of blunt chest injury, Adequate skill in regional analgesia prescription and management (Physical skills) Remembering to use protocol (Memory, attention, and decision processes)
Habit formation	Training	Staff will be encouraged to assess all potentially eligible patients systematically	Confidence in patient assessment skills, Confidence in skills needed for evidence-informed management of blunt chest injury, Adequate skill in regional analgesia prescription and management (Physical skills) Remembering to use protocol (Memory, attention, and decision processes)
Adding objects to the environment	Environmental restructuring, enablement	An icon will be added for the electronic medical record to flag to staff that patient is eligible for care bundle A pager will be setup to be able to contact staff responding to the care bundle	Remembering to use protocol (Memory, attention, and decision processes) Access to protocol (Environmental context and resources) Emotions relating to commencing new protocol (Emotion)
Restructuring the physical environment	Environmental restructuring, enablement	Equipment necessary for the care bundle will be placed in a location that ensures ease of access Equipment will be adequately labelled with instructions Additional equipment will be supplied to ensure adequate supply The protocol will be tested by staff to ensure ease of use	Remembering to use protocol (Memory, attention, and decision processes) Access to protocol, the protocol design, Access to equipment (Environmental context and resources) Emotions relating to commencing new protocol (Emotion)
Social Support	Enablement	Change champions will be chosen from each area who will receive extra training to be able to provide extra support	Social supports (Social influences)

Education: increasing knowledge or understanding, Persuasion: using communication to induce positive or negative feelings or stimulate action, Incentivisation: creating an expectation of reward, Training: imparting skills, Environmental restructuring: changing the physical or social context, Modelling: providing an example to aspire to, Enablement: increasing means/reducing barriers

#### Implementation strategies

##### Blunt chest injury care bundle video

A brief, entertaining, yet informative video was developed to address all seven of the intervention functions. A storyboard was developed to help guide the filming and editing process to ensure all intervention functions were represented and that the video targeted at the right audience. For example, to demonstrate intervention functions of modelling and reinforcement, the video features senior staff from each of the departments participating in the care bundle. Consent was sought before filming.

To demonstrate environmental restructuring, the video highlights the changes made to the environment including the electronic medical icon discussed below. The video was filmed using a smartphone and edited using easily accessible editing software by one of the team, keeping costs to a minimum. As there are two implementation sites, it was decided to have two videos. The videos are similar, but each has been adapted slightly to represent each site’s staff and the slight differences in implementation. The video was shared via email circulations from managers and educators and was presented during education sessions and the orientation of new staff.

##### Education sessions

Face-to-face education was chosen over online learning as this was preferred by stakeholders (participants in the survey); who reported that online training has been “overdone” in their workplace. Education sessions aimed to address the intervention functions: education, training, persuasion and modelling.

Education sessions for staff activating or responding to the care bundle were led by the emergency, surgical, intensive care educators at each site. They have been conducted regularly at multiple timeframes to suit the context and to increase participation. The educational sessions were varied according to the needs of the staff attending the session to address the different roles within the care bundle, for example, learning about regional analgesia. The video was presented in the sessions in an attempt to increase the engaging nature of the sessions and provide a memorable summary.

##### The electronic medical record icon

A function was added to the electronic medical record, allowing the activator of the care bundle to select an icon which highlights the patient to others. Restructuring the environment in this way aimed to make it easier for responding staff to identify patients on the care bundle and enable the initiation and use of the care bundle. “Responding” staff were told to expect this icon to find patients. Staff have been regularly reminded to activate the icon by the responding staff or the nurse consultants who audit in live time. Embedded within the icon are information prompts to progress the care bundle. The process for notifying and contact details for the responding team is different at each site; hence the information prompts are site-specific. This strategy aimed to address the intervention functions of enablement and environmental restructuring.

##### Change champions

Staff were hired as ‘change champions’ during the implementation; providing bedside training and additional support. Change champions can address all seven intervention functions; for example, they are in a position to provide education and training; and they can enable, reinforce and persuade staff directly. Change champions were employed or allocated as a formal part of their role. The educators, managers and clinical nurse consultants provide assistance and reinforcement for the clinical champions.

##### Audit/feedback

Audits and feedback were initiated to support education, training, persuasion and incentivisation functions, as they provide specific local data to staff on the effectiveness of the care bundle. The clinical champions and the nurse consultants have been responsible within their roles for ongoing monitoring of the activation and compliance of the care bundle. This aimed to allow for accurate and timely feedback to staff on the uptake of the care bundle and will allow opportunities for reinforcement, questions or corrections. Audit data on patients receiving the care bundle or requiring but not receiving the care bundle has been fed back to floor staff through change champions, educational sessions and emails to the heads of department.

## Discussion

This paper illustrates the use of validated frameworks of behaviour change to identify facilitators and barriers and select implementation strategies for the implementation of a *blunt chest injury care bundle*. This paper adds to researcher experiences of the prospective use of the TDF and Behaviour Change Wheel as a theory-based implementation strategy in emergency care [7, 12, 32, 36–39]. This paper provides a worked example of the use of the TDF and Behaviour Change Wheel used together systematically to identify facilitators and barriers and derive implementation strategies.

The facilitators and barriers identified were from eight out of 14 of the domains from the TDF, most commonly environmental context and resources, knowledge and skills. This finding is similar to other TDF based research in the emergency context [39–41]. Emergency departments are often busy, overcrowded, chaotic environments with pressures for flow of patients which may contribute to the importance of modifying the environment in this context [11].

The resulting implementation strategies were multi-fold to meet the identified facilitators and barriers to change. The videos featured staff relevant to the audience, which has been shown to be more effective [42]. Videos have been effective in promoting behaviour change in a variety of contexts such as teachers of children with autism [43] and for clinicians in the prescription of antibiotics [44]. However, video use in implementation needs to be concise and pitched at the right audience and level, otherwise may lead to mixed results [45].

Educational sessions were used as an intervention strategy due to stakeholders requests, and the literature supported this with no advantage reported in the effectiveness or the time taken for online learning compared to more traditional learning methods [46]. The education sessions were designed to be engaging and interactive using evidence-based techniques like case-based learning, practice and feedback to encourage maximum recall [47]. Furthermore, computer prompts have been demonstrated to have a small to moderate effect on behaviour [48]. Prompts such as the care bundle icon can be especially useful when staff are cognitively overloaded [49] such as in the emergency department.

Change champions have been successful in supporting change in healthcare [50]. Dedicated persons, such as locally-allocated change champions, are needed to drive implementation change and improve the fidelity of recommended protocols [51]; as they are more familiar with the local processes and in a better position to facilitate change [50]. They are also in a great position to help with audits and feedback which are supported by strong evidence as effective implementation strategies for change of care practices and clinical outcomes [52].

### Relevance to wider practice

The methods used in this study, prospectively applying the TDF and Behaviour Change Wheel to identify facilitators, barriers and implementation strategies can be applied to the implementation of a new intervention in the clinical environment. The use of a mixed-methods survey has been demonstrated as a feasible method to systematically identify facilitators, barriers and in extension inform implementation strategies. It may be a practical method to guide implementation in a more rapid but still systematic way. However, there was a lack of guidance in the frameworks for how to operationalise these strategies into an implementation plan. For example, other implementation frameworks in the literature recommend including facilitation as a necessary component of implementation [53]. Perhaps frameworks for implementation need to be used in combination, or a more comprehensive framework may be developed in the future.

### Limitations, strengths and future directions

There were some limitations to this study. Firstly, the survey response rate was lower than expected (45%). This was despite offering an incentive for completion and allowing time during work hours to complete the survey, which has been demonstrated to boost response numbers [20]. The overall estimate of staff obtained from departmental managers included part-time, casual and visiting staff, as well as those on maternity, annual and long service leave which may have exaggerated the actual numbers of staff available to participate. There is evidence to suggest that there is little relationship between response rate and nonresponse bias [1]; therefore, a lower response rate may not affect the validity of the study. Staff from all departments and levels of seniority completed the survey, enabling a representative sample. Secondly, it may be a limitation that we did not split the analysis of the sites at all levels of the process. However, the two hospitals are within the same area health service with many similarities including staff that work across both sites. Implementation differences were addressed at implementation in consultation with stakeholders.

The theoretical frameworks provided a systematic method for the identification of facilitators and barriers; however, our experience was of crossover between domains. These were resolved with a discussion between the authors. The authors agreed to categorise barriers or facilitators in one domain over another to aide in the integration and interpretation of the results while appreciating the potential interaction between TDF domains.

Using a mixed-methods approach was a strength of this study; however, more in-depth qualitative methods such as interviews were not used, and this may be a limitation. These methods were not used due to time constraints, with interview studies taking up to 24 months [22]. This may be revisited after evaluations of the implementation plan by investigating the uptake of the *blunt chest injury care bundle.* However, it may also be a novel way of using the Behaviour Change Wheel, that can lead to faster implementation.

Another strength was identifying the facilitators and barriers prior to implementation in the emergency and multidisciplinary context. Few studies were identified that used the TDF to inform the implementation strategy prior to implementation [33]. Translation of knowledge to clinical practice needs to be proactive to ensure that implementation is sustainable [54].

It will be necessary to evaluate the implementation; further research is underway to address this. It will also be necessary to evaluate the *blunt chest injury care bundle* for patient outcomes for a real-world evaluation of the care bundle, which will be undertaken with a retrospective observational study [55].

## Conclusions

Several facilitators and barriers may potentially impact the implementation of a *blunt chest injury care bundle*. Implementation strategies addressing these facilitators and barriers were designed using the Behaviour Change Wheel. The prospective design of targeted implementation strategies encourages the consideration of all the likely facilitators and barriers to change and is more likely to result in a higher sustained uptake. The implementation strategies identified in this paper were used to guide an implementation plan which will be evaluated post-implementation [see Additional file 2]. The authors aim for the results of that evaluation will lead to the development of an implementation resource for the implementation of the *blunt chest injury care bundle* in other contexts.

## Additional files

Additional file 1: Complete survey. This additional file is the full survey completed by participants. (PDF 93 kb)

Additional file 2: Implementation plan. This is a deidentified version of the implementation plan used for implementation at the sites. (DOCX 238 kb)

## Data Availability

The datasets generated and/or analysed during the current study are not publicly available due to lack of ethics committee approval but are available from the corresponding author on reasonable request.

## Abbreviations

APEASE: Affordability, Practicability, Effectiveness and cost-effectiveness, Acceptability, Side-effects or safety and Equity
BCT: Behaviour change technique
BCTTv1: Behaviour change taxonomy version 1
BCW: Behaviour Change Wheel
TDF: Theoretical Domains Framework
