# Supplementary material for: Designing strategies to implement a blunt chest injury care bundle using the behaviour change wheel: a multi-site mixed methods study
Source: BMC Health Serv Res. 2019 Jul 8;19:461. doi: 10.1186/s12913-019-4177-z (PMC6615309; doi:10.1186/s12913-019-4177-z)
Supplement: Supplementary file 1 — Complete survey. This additional file is the full survey completed by participants. (PDF 93 kb) [file 12913_2019_4177_MOESM1_ESM.pdf]

ChIP facilitators and barriers study

Thank you for taking the time to complete this survey. This survey is the first part of a multi-phased research project to implement and evaluate a Bundle of Care for patients with isolated blunt chest wall injury (ChIP).

This survey (phase 1) aims to identify the facilitators and barriers relating to the implementation of this new protocol at Wollongong and Shoalhaven Hospitals. Your responses will be anonymous.

Please ensure you have read the participant information sheet prior to continuing.

Thank you!

PLEASE NOTE YOU CAN CONTROL THE FONT SIZE FROM ZOOM BUTTONS IN TOP RIGHT-HAND CORNER OF YOUR SCREEN

You will have unlimited time to complete this survey, however, if you log out prior to completion, your responses will not be saved.

Participant information Sheet

Please take the time to read

[Attachment: "Participant information sheet v9_180317.pdf"]

Demographic Information

Which hospital do you work at?

- ◯ Wollongong Hospital
- ◯ Shoalhaven Memorial District Hospital
- ◯ Other
- ◯ Both TWH and SMDH

Thank you for completing our survey.

At this time, we are only surveying people who are current employees of The Wollongong or Shoalhaven Memorial Hospitals.

For more information please contact sarah.kourouche@sydney.edu.au

- ◯ Please close the browser window

What is your gender?

- ◯ Male
- ◯ Female

What is your age?

____
(in years)

What is your clinical department and role? (please select appropriate box)

- ◯ Anaesthetics - Doctor
- ◯ Surgical - Doctor
- ◯ Emergency - Doctor or Nurse
- ◯ Other e.g. specialist nurses, physiotherapists, non-clinical staff
- ◯ Respiratory - Doctor

Please select your current role

- ◯ Consultant / VMO
- ◯ Registrar / Fellow
- ◯ Resident / Intern

Please select your current role

- ◯ Consultant
- ◯ Fellow / Registrar
- ◯ Resident / Intern

Please select your current role

- ◯ Registered nurse (RN)
- ◯ Endorsed / Enrolled nurse (EEN/EN)
- ◯ Clinical Nurse Specialist (CNS)
- ◯ Staff Specialist / VMO
- ◯ Registrar / CMO
- ◯ Resident / Intern
- ◯ Senior nurse (Nursing unit manager, Nursing educator, Clinical nurse educator, Clinical nurse consultant etc)

Please select your current role

- ◯ Physiotherapy
- ◯ Switchboard
- ◯ Senior specialist nurse eg trauma, pain service, ICU liason etc

How long have you been in this role? Please answer in years

e.g. 6 months = 0.5

____
(Number response ONLY (in years))

Knowledge and Skills

Our hospital has a clinical protocol for the care of patients with blunt chest injuries (select one)

◯ Yes
◯ No
◯ Don't know

	Never	Very rarely	Rarely	Occasionally	Most of the time	Always
If I am caring for a patient with blunt chest injury, I know exactly what treatment to initiate	◯	◯	◯	◯	◯	◯

Please select the risk factors you feel are most likely to lead to deterioration for patients with blunt chest injuries

(Select all that apply)

☐ Diabetes
☐ Elderly
☐ Childhood asthma
☐ COPD / Chronic lung disease
☐ 3 or more rib fractures
☐ Myocardial infarction
(Select all that apply)

I am familiar with the term "care bundle" (select one)

◯ Yes
◯ No

A care bundle is: (select one)

◯ A guideline for the care of patients
◯ A group of key evidence-based interventions for a specific condition
◯ A pathway for care of a specific patient
◯ A recommendation for patient care

How important are the following interventions in the management of patients with blunt chest injuries?

(select your response on the scale for each intervention)

	Not Important	Slightly Important	Moderately Important	Important	Very important
Monitoring of arterial blood gases	◯	◯	◯	◯	◯
Maintaining Oxygenation with SpO2 above 95% (or appropriate to lung condition)	◯	◯	◯	◯	◯
Regular deep breathing and coughing exercises	◯	◯	◯	◯	◯
Early analgesia	◯	◯	◯	◯	◯
Regular multi-modal analgesia	◯	◯	◯	◯	◯
Patient controlled analgesia (opioid)	◯	◯	◯	◯	◯
Multidisciplinary approach with doctors, nurses and allied health professionals involved	◯	◯	◯	◯	◯
Monitoring of chest x-rays	◯	◯	◯	◯	◯
Early chest physiotherapy in the ED	◯	◯	◯	◯	◯
Chest wall splinting while coughing/deep breathing	◯	◯	◯	◯	◯
Regular chest physiotherapy	◯	◯	◯	◯	◯
Frequent reassessment of respiratory function	◯	◯	◯	◯	◯
Regular Incentive spirometry (e.g. TRIFLO). Device with 3 chambers each with a ball, patient required to blow so that the balls rise.	◯	◯	◯	◯	◯
Early mobilization	◯	◯	◯	◯	◯
Surgical fixation of rib fractures	◯	◯	◯	◯	◯
Clinical protocols for analgesia	◯	◯	◯	◯	◯

Any further comments? ____________________
(Free text)

I am confident in my ability to accurately......................

(Select your response on the scale for each intervention)

	Strongly Disagree	Disagree	Slightly Disagree	Slightly Agree	Agree	Strongly Agree
Locate landmarks to listen to a patient's chest	○	○	○	○	○	○
Assess a patient's respiratory effort	○	○	○	○	○	○
Assess pleuritic pain	○	○	○	○	○	○
Interpret findings from my assessment of a patient's respiratory function	○	○	○	○	○	○
Describe findings from assessment of respiratory function	○	○	○	○	○	○
Instruct a patient in the use of Incentive spirometer (TRIFLO)	○	○	○	○	○	○
Monitor for deterioration in a patient with blunt chest injury	○	○	○	○	○	○
Set up high flow nasal prongs	○	○	○	○	○	○
Prescribe high flow nasal prongs	○	○	○	○	○	○
Titrate flow rates for high flow nasal prongs	○	○	○	○	○	○
Prescribe appropriate analgesia for blunt chest injury	○	○	○	○	○	○
Prescribe appropriate epidural analgesia for blunt chest injury	○	○	○	○	○	○
Prescribe appropriate oral opioid analgesia for blunt chest injury	○	○	○	○	○	○
Prescribe appropriate paravertebral block analgesia for blunt chest injury	○	○	○	○	○	○
Prescribe appropriate Patient Controlled Analgesia for blunt chest injury	○	○	○	○	○	○
Prescribe (including nurse initiated/standing orders) early opioid analgesia	○	○	○	○	○	○
Manage epidural analgesia for blunt chest injury	○	○	○	○	○	○

Manage oral opioid analgesia for blunt chest injury	○	○	○	○	○	○
Manage intramuscular opioid analgesia for blunt chest injury	○	○	○	○	○	○
Manage intravenous opioid analgesia for blunt chest injury	○	○	○	○	○	○
Manage paravertebral block analgesia for blunt chest injury	○	○	○	○	○	○
Manage Patient Controlled Analgesia for blunt chest injury	○	○	○	○	○	○

Any further comments? ______________________________

(Free text)

Select your response in relation to your experience with clinical protocols

	Never	Very rarely	Rarely	Occasionally	Most of the time	Always
I find it easy to remember when to activate new protocols	○	○	○	○	○	○
During my work day I think there are too many protocols to remember	○	○	○	○	○	○

Any further comments?

____
(Free text)

How important are the following environmental factors in helping you remember to use clinical protocols.......

Please select on the scale

	Not Important	Slightly Important	Moderately Important	Important	Very important
Having a guideline on the intranet/internet	○	○	○	○	○
A form with a checklist kept in the patient notes	○	○	○	○	○
Flyers around the department	○	○	○	○	○
Email reminders	○	○	○	○	○
Simple criteria for activation	○	○	○	○	○
Having equipment easily accessible	○	○	○	○	○
Sticker placed in patient notes	○	○	○	○	○
emR tick box or icon	○	○	○	○	○

Any further comments? ______________________________
(free text)

Please rate how important (or not important) the following factors are in improving your use of clinical protocols:

Select from the scale

	Not Important	Slightly Important	Moderately Important	Important	Very important
Knowing I will be spoken to by a superior if I do not follow it	○	○	○	○	○
Knowing I am being audited	○	○	○	○	○
Feeling that the protocol will make a difference to the patient	○	○	○	○	○
Other staff reminding me to	○	○	○	○	○
Identifying myself as a "change agent"	○	○	○	○	○
Being aware of risks associated with not following a protocol	○	○	○	○	○

Please add any further comments here

____
(free text)

You're halfway! Please keep going your answers are highly valuable.

How likely are the following factors going to prevent you using protocols?

	Not at all	Somewhat	Moderately	Very much so
Too busy / interruptions eg with other patients, in OT, phone calls etc	○	○	○	○
No access to computer to access protocol / document	○	○	○	○
No access to phone to page / answer pager	○	○	○	○
Under staffed	○	○	○	○
Inadequate training in protocol use	○	○	○	○
Can't find protocol when I need it	○	○	○	○
Protocol not clear	○	○	○	○
I have my own process / way of doing things	○	○	○	○

Any further comments? ______________________________
(Free text)

How important are each of the strategies below in helping you to use and adhere to protocols in your workplace?

Select response on the scale

	Not Important	Slightly Important	Moderately Important	Important	Very important
Support from supervisor / manager	◯	◯	◯	◯	◯
Step by step instructions	◯	◯	◯	◯	◯
Concise flowchart	◯	◯	◯	◯	◯
Localised place for all protocols	◯	◯	◯	◯	◯
Protocols kept electronically	◯	◯	◯	◯	◯
Protocols up as posters in clinical environment	◯	◯	◯	◯	◯

Any further comments? ______________________________
(Free text)

I am more likely to follow a new protocol if I have support from.......................

(Select your response on the scale for each support source):

	Strongly Disagree	Disagree	Slightly disagree	Slightly agree	Agree	Strongly Agree
My superiors	◯	◯	◯	◯	◯	◯
Medical staff	◯	◯	◯	◯	◯	◯
Nursing staff	◯	◯	◯	◯	◯	◯
My colleagues	◯	◯	◯	◯	◯	◯
The patient	◯	◯	◯	◯	◯	◯
The patient's family	◯	◯	◯	◯	◯	◯

Any further comments?

____
(Free text)

How important are the following educational supports in using a new protocol?

(Select your response on the scale for each intervention)

	Not Important	Slightly Important	Moderately Important	Important	Very important
A short course on the protocol	◯	◯	◯	◯	◯
An online learning program	◯	◯	◯	◯	◯
Help on the floor from senior staff	◯	◯	◯	◯	◯
An in-service on the protocol	◯	◯	◯	◯	◯
My colleagues reminding me	◯	◯	◯	◯	◯

Do you have other comments relating to educational support provided? Please provide detail ______________________

It is my role to ....

(Select your response on the scale for each option):

	Strongly Disagree	Disagree	Slightly Agree	Agree	Strongly Agree
Assess patients with blunt chest injury	◯	◯	◯	◯	◯
Refer patients with blunt chest injury to a physiotherapist	◯	◯	◯	◯	◯
Prescribe analgesia for patients with blunt chest injury	◯	◯	◯	◯	◯
Assess and recognise if a patient with blunt chest injury needs further analgesia	◯	◯	◯	◯	◯
Decide if a patient with blunt chest injury needs admission	◯	◯	◯	◯	◯
Identify and escalate a deteriorating patient with blunt chest injury	◯	◯	◯	◯	◯
Care for patients on the ward with blunt chest injury	◯	◯	◯	◯	◯
Mobilise patients with blunt chest injury	◯	◯	◯	◯	◯

Any further comments? ______________________________
(Free text)

Almost finished! Thank you for your persistence.

I am confident I........

(Select response on the scale for each option):

	Never	Rarely	Sometimes	Very often	Always
Can adequately assess a patient with blunt chest injury	○	○	○	○	○
Can identify the patients with blunt chest injury that are at higher risk of complications	○	○	○	○	○
Can recognise patients with blunt chest injury who are deteriorating	○	○	○	○	○
Will know when to escalate care in a patient with blunt chest injury	○	○	○	○	○
Know when a patient with blunt chest injury can be discharged home safely or needs admission	○	○	○	○	○

Any further comments?

____
(Free text)

Please select how important the following statements are to you in your professional role:

(Select one response for each option)

	Not important	Slightly Important	Moderately Important	Important	Very Important
I want to minimise my patient's risk of complications	○	○	○	○	○
I want to have my patients appropriately managed as soon as possible	○	○	○	○	○
I want to have have my patient go to the appropriate level ward	○	○	○	○	○

Any further comments? ______________________________
(Free text)

With regards to your work at the hospital, do you agree or disagree with these statements?

(Select your response on the scale):

	Strongly Disagree	Disagree	Slightly Disagree	Slightly Agree	Agree	Strongly Agree
I am interested in being involved in the development of new protocols	○	○	○	○	○	○
I am interested in being involved in the implementation of new protocols	○	○	○	○	○	○
Despite any barriers that may occur, I strive to provide the best quality, evidence based care	○	○	○	○	○	○
Patients with blunt chest injury are provided consistent and evidence based care	○	○	○	○	○	○

Any further comments? ______________________________ (Free text)

When using new protocols in my practice, I feel................

(Select a response for each):

	Not at all	Somewhat	Moderately so	Very much so
Calm	○	○	○	○
Secure	○	○	○	○
Tense	○	○	○	○
Regret	○	○	○	○
At ease	○	○	○	○
Upset	○	○	○	○
Anxious	○	○	○	○
Comfortable	○	○	○	○
Self-confident	○	○	○	○
Nervous	○	○	○	○
Jittery	○	○	○	○
Content	○	○	○	○
Worried	○	○	○	○
Joyful	○	○	○	○
Pleasant	○	○	○	○

What do you think makes you feel this way? ______________________ (Free text)

Please comment (free text)

Patients with blunt chest injury are at risk of developing complications such as pneumonia and with inadequate treatment have higher rates of morbidity and mortality. ChIP is a protocol that is used in other hospitals and reduces risk of pneumonia by 56%. It activates an early multidisciplinary response (like a trauma call) and initiates evidence based guidelines for patients with blunt chest injury around analgesia, oxygenation, admission, and allied health reviews.

The following questions relate to this protocol. This is the last, but VERY important question :)

If a new protocol is implemented in your hospital, that:

activates an early multidisciplinary response (like a trauma call) and prompts evidence based guidelines for patients with blunt chest injury

What impact do you think it will have on you and/or your patient with blunt chest injury on the following?

(Select your response on the scale for each option):

	Strongly Disagree	Disagree	Slightly disagree	Slightly agree	Agree	Strongly Agree
There will be improvement in patient outcomes	○	○	○	○	○	○
My workload will be greater	○	○	○	○	○	○
My work day will be easier	○	○	○	○	○	○
There will be no change to my work day	○	○	○	○	○	○
There will be easier access to admitting team review	○	○	○	○	○	○
Patient will get earlier analgesia	○	○	○	○	○	○
There wll be improved time to physiotherapy review	○	○	○	○	○	○
There will be improved time to medical review	○	○	○	○	○	○
There will be reduced length of time in the emergency department	○	○	○	○	○	○
The patient will have earlier referral to aged care	○	○	○	○	○	○
There will be overall improvement in patient care	○	○	○	○	○	○
There will be reduced time to having a clear patient plan	○	○	○	○	○	○
The health care process will be improved overall	○	○	○	○	○	○
There will be improved time to surgical review	○	○	○	○	○	○
There will be improved time to pain team review	○	○	○	○	○	○
There will be decreased access to equipment	○	○	○	○	○	○

Any further comments? ______________________________
(Free text)
